# Interrelations of Physical Fitness and Cognitive Functions in German Schoolchildren

**DOI:** 10.3390/children8080669

**Published:** 2021-07-31

**Authors:** Alina Drozdowska, Michael Falkenstein, Gernot Jendrusch, Petra Platen, Thomas Lücke, Mathilde Kersting, Kathrin Sinningen

**Affiliations:** 1 Research Department of Child Nutrition, University Hospital of Pediatrics and Adolescent Medicine, St. Josef-Hospital, Ruhr University Bochum, 44791 Bochum, Germany; luecke.thomas@ruhr-uni-bochum.de (T.L.); mathilde.kersting@ruhr-uni-bochum.de (M.K.); kathrin.sinningen@ruhr-uni-bochum.de (K.S.); 2 ALA Institute, 44805 Bochum, Germany; falkenstein@ala-institut.de; 3 Department of Sports Medicine and Sports Nutrition, Ruhr University Bochum, 44801 Bochum, Germany; gernot.jendrusch@ruhr-uni-bochum.de (G.J.); petra.platen@ruhr-uni-bochum.de (P.P.)

**Keywords:** physical fitness, physical education, cognition, physical activity, school-aged children, coordination

## Abstract

This study investigated the relationship between different levels of physical fitness and cognitive functions in boys and girls. Schoolchildren from a comprehensive school in Germany (*n* = 211, 39% girls, 5th and 6th grade) attended regular or sport-focused classes with different numbers of physical education (PE) classes per week (3 vs. 5–6 h). Performance of physical fitness was tested according to endurance, strength, speed, coordination and flexibility. Four computerized instruments (switch task, 2-back task, Corsi block-tapping task and flanker task) were used to test cognitive functions. Additional predictors, sex, age, PE class, Body Mass Index and physical activity, were included in analyses. The results showed that physical fitness was associated with improved attention and memory functions in children, although the associations were mostly small. After Bonferroni correction, mainly coordination was related to improved cognition. Physical activity, i.e., step counts, PE class and sex were associated with specific cognitive outcomes. These findings may be important for effective health promotion, and supporting children’s education in the school environment. Sex-specific physical activities in school could potentially lead to greater cognitive benefits in children. Randomized trials are needed to replicate these results.

## 1. Introduction

There is growing interest in fitness-related research on children. Scientific attention is focused not only on fitness level, including muscle strength, cardiorespiratory endurance and motor coordination, but also on fitness benefits [1,2]. Many studies have suggested that physical fitness in youth and cognitive skills in later life are closely related [3,4,5]. It was found that primary schoolchildren with higher fitness skills have an advantage over their peers with lower fitness performance in specific cognitive functions [6,7], which can lead to better academic performance [4,8,9]. Studies on children have shown that cardiorespiratory fitness is related to inhibition and cognitive flexibility [10], as well as memory ability [11] and selective attention [12]. Equally important is the possibility that skills and behavior acquired at a young age may persist into adulthood [13]. Results from a Finnish study have demonstrated that a physically active childhood (age 6–12 years) was associated with faster cognitive response in midlife (age 34–39 years) [14]. The positive effects of physical fitness stimulated by physical activity can be explained by long-term adaptation processes of the brain. Physical activity can influence the brain’s structure [11], including neurogenesis [15], and stimulate brain activity [16]. 

Despite the positive effects of physical fitness mentioned above, there is evidence that physical activity and fitness skills are inadequate in children and decrease with age [17,18,19]. There are differences in physical fitness across the countries. Lower coordination skills such as balance and side jumping were observed in Portuguese children compared to children from Finland or Belgium [18] and locomotor skills of Belgian children were reported to be better than those of children from the USA [19] or Australia [20]. German children outperformed Omani children in a sprint over 20 m but not in coordinative tests [21]. Given these country-specific differences in children’s fitness skills, it is vital for effective health promotion to recognize the strong correlation between specific fitness and cognitive skills. To date, the relationship between various aspects of fitness and cognitive performance in schoolchildren has been largely based on observational research, particularly with regard to endurance performance. Infrequently conducted randomized studies predominantly used mixed training programs to examine the effects of physical activity on cognition [5,8].

Moreover, it is important to focus on a school environment to promote children’s physical activity and healthy development. Results of the representative cross-sectional survey of children and adolescents in Germany indicated a positive correlation between children’s sports participation and an activity-friendly environment (playgrounds with playing or sports facilities, sports fields, swimming pools, parks/green spaces) [17]. Once children have the opportunity to be active outdoors or participate in organized sports, fitness skills seem to increase [22]. Given the growing interest in schools as a setting for the promotion of physical activity, it may be beneficial to introduce more physical education (PE) into regular curricular lessons at school [23]. As a result, no loss of academic performance is expected; on the contrary, it seems to cause an improvement [24].

The interplay of physical fitness and cognitive abilities in German schoolchildren has rarely been studied [5]. None of the studies compared physical fitness and cognitive performance of German children aged 10–12 years, taking into account other characteristics of the selected study population [9,21,25,26]. Consequently, the objective of this study was to investigate the relationship between physical fitness and cognitive functions in 10–12-year-old boys and girls using validated German fitness tests and specific cognitive tasks, considering additional predictors, namely, sex, age, PE class, BMI and physical activity. It has been hypothesized that better fitness skills are related to better cognitive performance, but the strength of the association between specific fitness skills and specific cognitive performances varies.

## 2. Materials and Methods

### 2.1. Study Design

This baseline data set represents a subset of the randomized intervention trial CogniDROP (Cognition, Drinking Observation and Physical Activity), which focused on the short-term effects of water intake on specific cognitive functions in schoolchildren and was used for this secondary analysis. The study design has been described elsewhere [27]. Intervention procedures and randomization data were neglected in this assessment. Data of cognitive performance and physical activity were collected between October 2018 and January 2019, excluding school holidays. In addition, physical fitness tests were carried out independently on four testing days in November 2018 in no particular order of Participants. Cognitive and fitness tests, which were integrated into the usual school procedures, were performed once within the class community. Cognitive tests were always performed before the lunch break (at 12:20 p.m.), while the fitness tests were carried out on separate days during the school day (8:00 a.m.—3:00 p.m.). The Ethics Committee of the Faculty of Sport Science of Ruhr University Bochum has approved the study protocol (EKS V 22/2018, approved on 4 September, 2018). All procedures were performed in accordance with the Helsinki Declaration (Trial registration: DRKS00017115).

### 2.2. Participants

Schoolchildren of the 5th and 6th grade (14 classes, 400 children) from a comprehensive school in Gelsenkirchen (North Rhine-Westphalia) in Germany were invited to participate. Exclusion criteria were diagnosed learning disabilities. Participating children and their parents/guardians provided written informed consent. This secondary public school ranges from 5th to 13th grade and is accessible to children of all educational backgrounds. Girls and boys have classes together, including PE. In this particular school, each grade consists of regular and sport-focused classes with different numbers of PE classes per week. At that time, there were eight regular classes (r-PE) with 3 h of PE a week and six sport-focused classes (s-PE) with 5 h of PE a week in the 5th grade and 6 h in the 6th grade. Requirements for admission to an s-PE class are above-average fitness skills that are tested (Motor Test of North Rhine-Westphalia in Germany, NRW Test [28]) five months before entering school in the 5th grade. Only children with above-average fitness skills can apply for the s-PE class. Children applying for r-PE are not tested. Apart from this, the same PE-educational goals (including ball games, gymnastics, athletics, dance and swimming [29]) based on the guidelines for the school sport in Germany [30] are pursued in both groups (s-PE and r-PE).

### 2.3. Fitness Tests 

**Figure 1:**
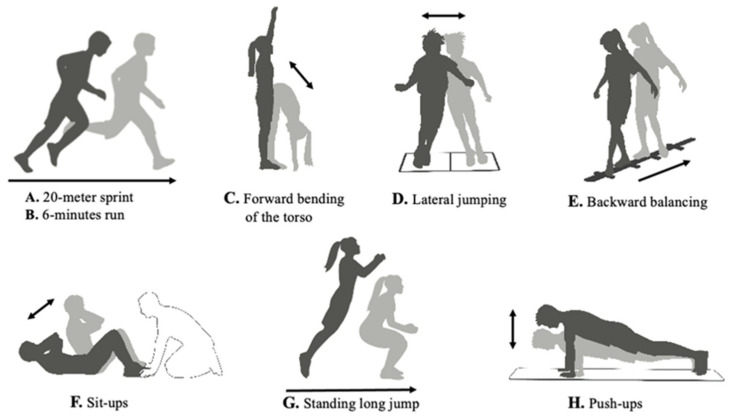
Physical fitness tests via the German Motor Test 6–18 (DMT 6–18). Performance: (**A**) Speed via 20-m sprint; (**B**) Endurance via 6-min run; (**C**) Flexibility via forward bending; (**D**) Coordination of the jumping; (**E**) Coordination of the backward balancing; (**F**) Strength of the abdominal muscles via sit-ups; (**G**) Strength of the lower extremities via jumping ability; (**H**) Strength of the upper extremities via push-ups; DMT, Deutscher Motorik-Test.

Children’s physical fitness was assessed by the German Motor Test 6–18 (Deutscher Motorik-Test 6–18, DMT 6–18) for children and adolescents between 6 and 18 years of age [28]. The tests were conducted at school (four separate days in November) by trained instructors of the Department of Sports Medicine and Sports Nutrition, Ruhr University Bochum in no particular order of classes. The validated DMT consists of eight subtests according to five fitness skills: speed (1: sprinting), endurance (2: running); torso flexibility (3: forward bending), coordination (4: lateral jumping and 5: balancing backwards) and strength (6: sit-ups, 7: standing long jump and 8: push-ups) (Figure 1). Fitness performance was expressed based on the standards of the DMT as an age- and sex-specific Z-score for each subtest of the five fitness skills. Z-score was defined as deficient (<92 points), below average (92–97 points), average (98–102 points), above average (103–108 points) and excellent (>108 points). Total Z-score was the average score of all fitness skills.

*Speed*. To test the body’s endurance strength and speed, a 20-m sprint was performed twice. The best time was assessed with a stopwatch. The sprint began after a signal with an upright posture at the start line.

*Endurance*. The aerobic endurance capacity of the cardiovascular system was determined by the results of running performance in the sports hall marked out with pylons. The total distance of a 6 min run was recorded.

*Flexibility*. Forward bending of the torso was performed to test the muscle flexibility of the torso and legs. Standing on a bench, the children bent slowly forward to reach the bench with their hands. The distance to the bench was recorded.

*Coordination*. Lateral jumping was performed twice with both legs over the center line of a field as quickly as possible for 15 s. Number of jumps was measured. The best result was used for the evaluation. Backward balancing under precision constraint was examined on beams of 300 cm length and 3, 4.5 and 6 cm width. All backward steps without touching the ground were evaluated.

*Strength*. The strength of the chest, abdominal, upper and lower extremity muscles was tested through sit-ups, jumping forward and push-ups. The number of sit-ups achieved in 40 s was counted, with the trainer holding the feet. Standing long jump was carried out with both feet. The distance from the starting line to the heel after landing was measured. Push-ups were counted within 40 s.

### 2.4. Cognitive Assessment

**Figure 2:**
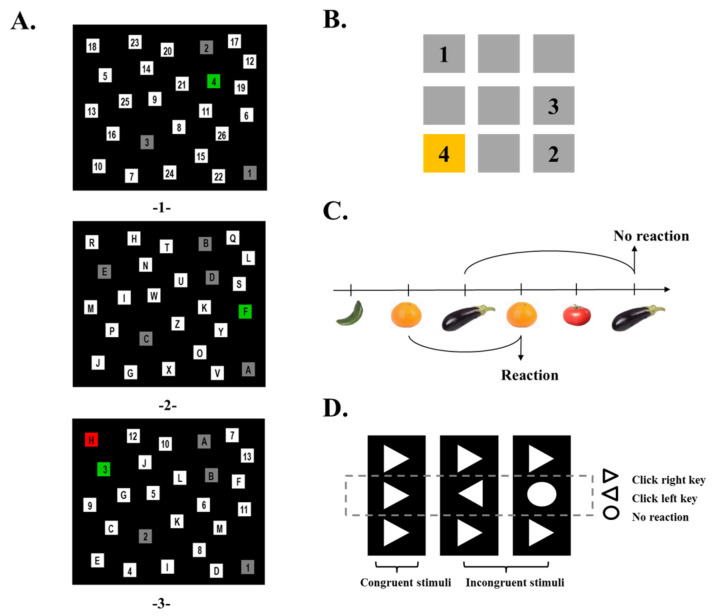
Cognitive task. (**A**). Visual attention and switching abilities between two targets: numbers and letters, measured by **task switching**. The task is comprised of three sections. 1. First section, numbers (non-switch). Numbers had to be clicked in ascending order with the mouse cursor. 2. Second section, letters (non-switch). Letters from A to Z had to be clicked alphabetically. 3. Third section, consecutively switching between numbers and letters in ascending order (i.e., 1-A-2-B-3-C…13-M). The target turned red if the answer was incorrect or green if the answer was correct. (**B**). Visual-spatial memory measured by the **Corsi block-tapping task**. Nine blue squares appearing as stimuli on the screen changed the color in a spatial random order. Three to six block sequences were displayed and had to be repeated in the same order. (**C**). Working memory updating measured by a **2-back task**. Fruits and vegetables were displayed on a computer screen. A predefined key had to be pressed when the current image was the same as the image two trials back. (**D**). Inhibitory control measured by **flanker task**. Directional response to the stimuli: congruent flankers and incongruent flankers were needed by pressing a defined computer key.

Cognitive functions were assessed using computerized assessment tools which were assembled in cooperation with the ALA Institute in Bochum, Germany (Figure 2) and adapted to the study conditions. A detailed description of the assessment has been published previously [27]. First, the cognition of all sixth graders was tested, then that of the fifth graders (with the exception of one 6th grader who was tested in January). The speed and accuracy of the responses in the task switching, Corsi block-tapping task, 2-back task and flanker task were measured. The testing time was around 30 min. Incomplete cognitive measurements were recorded as missing data.

*Task switching*. The task was performed to assess the ability to shift attention between two targets: numbers and letters. First, the children were asked to click numbers from 1 to 26 in ascending order as quickly as possible (Figure 2A). The second part was carried out with letters from A to Z in a similar way (alphabetical). Finally, the children had to switch between both targets and alternately click numbers and letters from 1-A to 13-M in the correct order (switch trial) (i.e., 1-A-2-B-3-C…13-M). If the wrong reaction was selected, the target turned red and if the correct reaction was selected, it turned green. The reaction time (RT) of the selective visual search for numbers and letters, as well as for switch trial in seconds were measured. Switch costs included results of all three parts and reflect the time extension by task switching, calculated as previously described [27,31]. The switch costs could only be calculated if all three tasks were successfully completed within 3 min.

*Corsi block-tapping task*. Visual-spatial memory ability was tested (Figure 2B). Nine blue squares appearing as stimuli on the screen changed color in a spatially random order. Three to six block sequences were displayed and had to be repeated in the same order. Stimuli recall of 12 block sequences had to be reproduced with increasing lengths: 3-, 4-, 5- and 6-boxes, three times each. The outcome measure was the longest remembered path, correct immediate block span (number of correctly reproduced sequences) and a score calculated for each sequence length. The awarding points: a minimum of one point for the three-box sequence up to a maximum of four points for the six-box sequence. More points indicate better visual–spatial memory.

*2-back task*. The task was performed to assess the ability of working memory and stored information updating. Images with fruits and vegetables were sequentially presented in the center of the screen (Figure 2C). Participants had to press a defined computer key when the current image matched the image from two trials back. In total, 21 of the 106 images were targets (correct trials). Outcome measures were the ratio of false alarms (response to wrong trial), count of all correct events (total number of correct responses and the number of no responses to wrong trial) and RT calculated only for correctly responded trials [27].

*Flanker task*. Inhibition was assessed by responding to the directional targets. The task consisted of 102 trials: 35 congruent flankers, 35 incongruent flankers and 32 no-go target stimuli (Figure 2D). When the stimuli were congruent, the right index finger should press a defined computer key. When the stimuli were incongruent, the left index finger should press a defined computer key. The participants were asked to suppress a reaction to the circle. The dependent variables were the mean RT of correct responses to congruent (RT Cong) and incongruent (RT ICong) stimuli, the count of false alarms in no-go trials, ratio of incorrect responses to incongruent (Ratio False ICong) and congruent (Ratio False Cong) stimuli. Responses outside a time window of 1200 ms after stimulus onset (an impulsive reaction) were considered as missing.

### 2.5. Additional Predictors

Other measures such as physical activity and Body Mass Index (BMI) were included as predictor variables for the analysis of the selected study population (PE class, sex and age). 

#### 2.5.1. Physical Activity

Physical activity was measured on PE-free days at school using the ActiGraph GT3X accelerometer (ActiGraph, Pensacola, FL, USA) worn on the wrist. This validated measuring instrument is widely used by researchers and a reliable tool for measuring physical activity intensity (sedentary, light, moderate and vigorous) and steps taken [32]. Information based on high-resolution raw acceleration data during three-axis movement recording is calculated algorithmically for use. In this study, the level of participants’ physical activity was measured for a 24 h period, starting one day before the study day (from 1:00 p.m.) until completion of the cognitive tests on the study day (to 1:00 p.m.). Additional measurement of physical activity was recorded between 8:00 a.m. and 1:00 p.m. on the test day. Measurement expressed in step counts was complete, if wear time was 100% per day. The recommendations in Germany for physical activity in children and adolescents are a daily value of at least 12,000 steps [33]. Measurement of the physical activity intensity was neglected in this assessment.

#### 2.5.2. Body Mass Index

Body weight and height (lightly clothed, without shoes) were measured as part of the fitness tests using a Seca 862 digital scale (the accuracy of measurement ± 50 g up to the weight of 150 kg) and a Seca 213 mobile stadiometer up to a height of 205 cm (Seca Corporation, Hamburg, Germany). BMI was calculated as the body weight (in kg) divided by the square of the height (in m). According to the Centers for Disease Control and Prevention (CDC) norms, a BMI above the 90th percentile for the average age of the study population was considered as overweight (~22 kg/m^2^) [34].

### 2.6. Statistical Analyses

Data were analyzed using SPSS statistical software package version 25.0 (IBM Corp., Armonk, NY, USA). Significance was accepted at the α-level of 0.05 (α ≤ 0.05). A Bonferroni correction was applied to consider multiple testing of the 15 cognitive outcomes (significance level: α/15 (α ≤ 0.003). Data were preliminarily checked for normality using the Shapiro–Wilk test. A descriptive analysis of the characteristics was carried out by non-parametric tests (Chi-squared Test or Fisher’s Exact Test) for dichotomous categorical variables (PE class, grade and sex). Parametric data from two independent PE classes were run by Mann–Whitney U Test for non-normally distributed data or *t*-test for normally distributed data to compare means (age, BMI, physical activity and physical fitness). Cognitive parameters were the primary outcomes of the analysis. Bivariate correlations (Spearman’s rank correlation coefficient) were performed to test the relationship between different fitness skills and specific cognitive parameters.

Additionally, linear regression models were conducted to determine the relevant predictors (BMI, sex, age, PE class and step counts) of dependent variable fitness skills. Next, stepwise regressions with backward elimination considering all predictors (physical fitness, PE class, BMI, sex, age and step counts) were performed to test the association with each cognitive outcome.

## 3. Results

### 3.1. Participants

The final sample size was 211 children, who took part in the cognitive tasks on the study day, finished all fitness tests and met the inclusion criteria. The participants’ characteristics are presented in Table 1. Significantly more boys than girls participated in the study (*p* = 0.002). There was no significant sex difference in BMI, neither within the PE classes nor overall (*p* > 0.05). According to the CDC norms, out of 211 children, 52 (24.6%) were overweight, including 23 girls and 29 boys (five children in the s-PE).

### 3.2. Physical Fitness

In general, children from the s-PE (5–6 h of PE a week) scored better in fitness tests than children from the r-PE (3 h of PE a week). Children in the r-PE scored below average on the fitness tests, with a score of 97 according to DMT standards, while s-PE scored excellent, with an average of 109 (Table 1). Regarding the five categories of fitness skills, the s-PE scored significantly better in all categories compared to the r-PE. In the endurance test the achieved score was the lowest. Only 41 children scored above average in the 6 min run (37 children from s-PE). The performance in coordination was the best. Of 211 children, 52.6% children (89 boys and 22 girls) performed excellently. Considering sex-specific differences, boys outperformed girls in fitness performance (*p* < 0.001, boys scored 104 ± 7.6 and girls scored 99 ± 8.5).

Table 2 summarizes the findings of the regression analysis with the total Z-score of DMT 6–18 as a dependent variable. Overall, the predictors explained about 50% of the variance in fitness performance. In a complementary model, the PE class made a statistically significant contribution to the model (R^2^ = 0.643, *p* < 0.001) and accounted for an additional 14% of the variance in DMT. For the total Z-score of DMT, BMI, age, sex and step counts were significant predictors. The strongest association was observed for BMI. With higher BMI and age, the impairment of general fitness skills increased. An additional analysis of the Spearman correlations showed that fitness skills decrease differently with age (significant for endurance: *p* = 0.03, r = −0.15; strength: *p* = 0.004, r = −0.20 and coordination: *p* = 0.003, r = −0.21; not significant for speed: *p* = 0.16 and flexibility: *p* = 0.19). 

The correlations within all subtests of the DMT were positive, large and significant (r > 0.5, *p* < 0.01) except the associations between flexibility and other skills, which were weak to moderate (0.1 < r < 0.5, *p* < 0.05).

### 3.3. Fitness Skills and Cognitive Functions

Bivariate correlations between the physical fitness total Z-score, subtests according to speed, endurance, flexibility, coordination and strength, and the cognitive variables were calculated. When a significant association was found, improved cognitive performance was always associated with an increased score in fitness skills (Table 3). After applying the Bonferroni correction (corrected *p*-value 0.003), there were still significant associations between coordination, speed and the total Z-score with visual-spatial memory in the Corsi block-tapping task (*p* ≤ 0.001), as well as RT to congruent trials in the flanker task (*p* ≤ 0.001). The association between the RT in the switch task and the RT in the 2-back task remained significant only for coordination.

### 3.4. Cognitive Functions and Additional Predictors

The results of the stepwise regression with backward elimination between selected cognitive functions and fitness skills taking into account additional predictors (sex, age, PE class, BMI and physical activity via step counts) are shown in Table 4. Overall, the predictors in total including the fitness skills explained around 3–19% of the variance in cognitive performance. Fitness skills were the strongest predictors for the switch and Corsi block-tapping tasks. Sex was the strongest predictor of accuracy in the 2-back and flanker tasks, indicating fewer errors and more correct events for girls compared to boys. A significant association between the PE class was observed for at least one parameter in the 2-back and flanker task, suggesting better reaction speed (flanker task) and accuracy (2-back and flanker task) in the s-PE. The accelerometer-based physical activity on the test day, expressed as step counts between 8:00 a.m. and 1:00 p.m., showed a positive association with reaction speed and accuracy (a higher number of steps on the test day was associated with a shorter visual search in the switch task and shorter RT in the 2-back task). Fewer false alarms were observed in the 2-back task and flanker task with an increasing number of steps on the test day, and concurrently more correct events in the 2-back task. The step counts for the 24 h period were the most frequently excluded predictor in models (195 children wore an accelerometer over the 24 h period). Age showed a positive association with the results of attention tasks (false alarms in the flanker task and visual search in the switch task, except for letters), suggesting better results with increasing age, while BMI was a weak predictor for the Corsi block-tapping task. After Bonferroni correction, however, there was no longer any significant interaction between fitness skills and cognitive functions, with the exception of coordination and RT in the switch task, as well as no associations between age, BMI, step counts or PE class and cognition, while sex was still the strongest and most significant predictor of outcomes in the flanker task.

## 4. Discussion

The purpose of this study was to examine associations between physical fitness and cognitive functions among school-aged children, considering additional predictors. The main finding was that a few significant relationships between the diverse fitness skills and the 15 cognitive parameters were found, suggesting beneficial associations of physical fitness with cognition as described elsewhere [4,6,25]. Several other predictors, mainly sex, were also relevant for specific skills. However, only a small part of the variability in cognitive differences could be explained by the models. 

### 4.1. Physical Fitness and Cognitive Functions

The findings of this study support the assumption that fitness skills may be beneficial for cognitive functions, similar to other studies with children of the same age [4] and younger children [35]. However, the identified associations between fitness and cognitive skills were mostly weak, similar to the results of other researchers [6]. Fitness skills are believed to have a stronger influence on cognition in younger children, so that the influence becomes less evident with increasing age [9,36]. On the other hand, more complex fitness skills with cognitive demands such as planning or controlling movement showed greater effects [37]. It could also be assumed that other lifestyle factors play a role in this context [38]. Since all results of the DMT subtests correlated strongly with each other, the results should be interpreted with caution. 

Interestingly, cognitive effects of both motor (body coordination) and cardiovascular training (running activities) on working memory were shown in children aged 9 to 10 years after 10 weeks of intervention [25]. However, the specific training sessions could only improve the respective fitness ability, coordinative training could only improve coordinative abilities and running activities could only improve cardiovascular performance. Accordingly, the authors concluded that the adaptive patterns of the children’s brains differed after coordinative and cardiovascular interventions, which is comparable to the results in adults [39]. Both skills seem important as both increase cognitive effects through increased cerebral blood flow and changes in the structure of the brain [40]. The cerebral stimulation through various training programs improves the plasticity of the brain in different regions with specific cognitive demands [40,41,42]. 

Regarding the various fitness skills in the current study, coordination was found to have the most frequent and highest correlations with cognitive functions, which is in line with another study [4]. Although the cognitive demands on coordinative performance seemed to be higher than for endurance and muscle strength, it was surprising that the relationship between coordination and cognition differed only slightly from other skills. A previous study with 10–12-year-old children found that even balancing showed no correlation with cognitive functions, while locomotor skills (forward roll starting with a jump, running forward and sideways) were only linked to working memory in the 2-back task but not inhibitory control and cognitive flexibility in the flanker task [6]. The discrepancies between the studies could be explained by variability in fitness measurements and possibly different levels of specific fitness skills in children. On the other hand, coordinative abilities of high cognitive demands, such as dribbling, throwing and catching a ball, show a stronger influence on the cognitive processes in schoolchildren [6,37] and kindergartners [43]. It can be assumed that coordinative skills tested in this study population were less challenging compared to other studies because the coordinative performance was the best and almost 53% children achieved excellent results according to DMT standards. Consequently, more sophisticated coordinative tests could produce stronger associations. 

Of all the DMT subtests, the endurance capacity of the cardiovascular system (the results of a 6 min run) was the lowest and only associated with the memory tasks, while the speed and strength of endurance (via sprint performance) showed associations with at least one parameter in each cognitive task. Others, who used the same fitness tests but examined a younger study population (age from 6 to 8), showed two conflicting results compared with the findings of this study: (1) a strong relationship between aerobic endurance and academic skills such as language understanding and mathematical thinking; (2) no correlations between sprint and academic skills [9]. This can be explained by special differences between the two studies. Children in this study showed significant losses in endurance development with age (from 10 to 12 years) and stagnation in sprint performance, in contrast to younger children in the other study who showed significant progress in sprinting and stagnation in endurance performance (from 6 to 8 years). According to Dumontheil [44], the period from childhood through adolescence is the time of changes in the maturation and functioning of the brain with a relationship to working memory and inhibitory control. Therefore, older children are likely to need more demanding fitness tasks to stimulate specific regions in the brain, while younger children will continue to benefit from simple challenges [36]. Páez-Maldonado et al. [12] showed that calculating cardiorespiratory fitness in children (10 to 12 years of age) using the maximum oxygen consumption (VO_2_max) formula provides additional information on the relationship with cognition. The authors documented a significant relationship between selective attention or concentration and sprint performance or VO_2_max (correlation coefficient of sprint performance r < 0.30, correlation coefficient of VO_2_max r > 0.40). Looking ahead, using more specific fitness tests in older children could contribute to the understanding of associations.

Inconsistent evidence of a relationship between muscle strength and cognitive ability has been found in a previous study [45]. There is only evidence of muscle strength benefits in children for bone health and a reduced risk of metabolic syndrome. More specifically, no correlation was found between lower limb strength via jumping performance and attention [12], as well as inconsistent interaction with academic skills [9], memory, inhibition control and shifting [4]. Furthermore, no association between abdominal or chest muscle strength and inhibition control was found [46]. Interestingly, a neuroimaging study in children has shown an association between muscle strength and two regions in the white matter of the brain, which in turn were related to academic ability [42]. However, the results of this study were able to suggest benefits of muscle strength for accuracy in memory tasks as well as for RT in the flanker task. Consequently, the results suggest that muscle strength may have an impact on cognition, and further randomized trials of children exploring this relationship may support these findings.

Finally, the performance of forward bending in this study showed the smallest, if any, association with cognition. In terms of comparing results, similar studies are rare. There is only one study of flexibility and cognition in children that showed no association between flexibility and inhibitory control [46]. More studies are needed to draw the necessary conclusions.

### 4.2. Additional Predictors

Physical activity (the number of steps on the test day, from 8 a.m. to 1 p.m.) was associated with accuracy and response time in all tasks in the regression models. It is possible that memory and attention benefited from the short-term effects of physical activity in the morning due to improved neurological stimulation of brain activity [47]. Although this variable was not the strongest in the models, it was possibly relevant for the cognitive variability, and could, together with structured and more frequent PE in school, induce a possible mediating long-term mechanism via physical fitness [4]. Taken together, PE in school and a higher level of habitual activity could help to strengthen existing skills or prevent the progressive loss of fitness skills with age, which has been reported elsewhere [17,18,19,20]. In this context, school seems to be an important environment to increase children’s physical activity and fitness.

Furthermore, the finding that sex was most often among the predictors for specific cognitive outcomes in the multivariable models is supported by the literature, where specific differences have often and convincingly been reported [48,49]. Specific training of fitness skills could reduce these cognitive differences between girls and boys [50]. Despite the significant correlations between the variables in the present evaluation, it is important to point out that other factors could also influence cognitive functions (e.g., nutrition, and psychological and socioeconomic factors [27,38]). Thus, it is impossible to consider different influencing factors separately.

### 4.3. National Context

There were other important findings outside of the primary results in this study which are possibly interesting for the regional differences within Germany. The results showed that overweight prevalence was higher in this sample (almost 25% of the children) than in German children for this age group (about 20% of children between the ages of 11 and 13 are overweight in Germany) [17]. In addition, the findings of the study indicated a high prevalence of inadequate fitness skills in this sample, especially in the r-PE group. These sample characteristics may have their origins in socio-economic and cultural disparities, such as lifestyle practices. However, we cannot definitively explain these results.

### 4.4. Strengths and Limitations

The present study has several strengths. First, four well-established computerized tasks to test various cognitive functions and validated tests were used to examine physical fitness in German schoolchildren. Second, physical activity for 24 h were measured using a scientifically recognized accelerometer. Moreover, various predictors such as physical education, Body Mass Index, age and sex were included in the analysis. Additionally, the cognitive abilities of children were tested in the natural school environment. The non-PE days were chosen for cognitive testing to avoid any confounding effects of acute PE.

Nevertheless, we acknowledge some study limitations. Fewer girls than boys participated in the study, especially from the s-PE group. The inclusion requirement for sports-focused classes probably had an impact on the sex distribution in PE classes. The physical fitness tests were measured at different times of the day, which could affect fitness performance. When measuring physical activity with accelerometers, collecting data over several days would better reflect the habitual activity. However, the implementation was not possible in the school, as electronic devices are not allowed during physical education for safety reasons. Psychological, biological and environmental factors were not included in this study which could have complemented the results.

## 5. Conclusions

In conclusion, current results suggest that there are mostly weak associations between physical fitness and cognitive functions. Children with better fitness skills show better attention and memory performance, while coordinative skills seem to be more relevant in this context than other fitness skills. Sex can also contribute to the cognitive differences. Furthermore, differences in physical education curricula and physical activity may affect differences in fitness competence and cognitive performance. Particular attention should be paid to sex inequality to ensure that fitness and cognitive skills are promoted in both girls and boys. These findings are of great importance for effective health promotion.

## Figures and Tables

**Table 1:** Sample characteristics *n* = 211.

	s-PE	r-PE	*p*-Value
*n*	90	121	
Grade 5 *n* (%)	46 (51.1)	54 (44.6)	
Grade 6 *n* (%)	44 (48.9)	67 (55.4)	
Boys *n* (%)	73 (81.1)	55 (45.5)	
Girls *n* (%)	17 (18.9)	66 (54.5)	
Age in years (month)	10.8 ± 0.7 (135)	11.1 ± 0.8 (138)	0.020 (0.058)
BMI	18.0 ± 2.4	21.3 ± 4.7	<0.001
DMT 6–18 Total Z-score ^†^	109 ± 4.4	97 ± 7.3	<0.001
Speed	111 ± 6.7	100 ± 11.4	<0.001
Endurance	101 ± 7.8	86 ± 8.2	<0.001
Flexibility	101 ± 9.6	97 ± 11.0	0.003
Coordination	115 ± 6.1	103 ± 9.4	<0.001
Strength	109 ± 5.4	96 ± 8.1	<0.001
Step Counts 24 h	16113 ± 2996	15223 ± 2720	0.042
Step Counts 8 a.m.–1 p.m.	4782 ± 744	4745 ± 819	0.739

Descriptive analysis of the dichotomous categorical variable (PE class, grade and sex) by non-parametric and parametric tests (frequency distribution, mean ± standard deviation); ^†^ Total Z-score is the DMT 6–18 average score of all eight subtests (*n* = 211); *p*-values based on Mann–Whitney *U* Test for non-normally distributed data or *t*-test for normally distributed data; Abbreviations: BMI, Body Mass Index; DMT, Deutscher Motorik-Test; r-PE, regular physical education classes; s-PE, sport-focused physical education classes.

**Table 2:** Linear regression model with the total Z-score of DMT 6–18 as dependent variable and adjusted predictors: BMI, age, sex and step counts in 195 children.

Total Z-Score		Coefficients	
Predictors	Unstandardized	Standardized	Std. Error
Constant	143.725 ***		
BMI	−1.193 ***	−0.601	0.101
Age	−0.129 **	−0.142	0.047
Sex	−4.528 ***	−0.268	0.864
Step Counts 24 h	0.0004 **	0.135	0.0002
Observations	195		
R^2^	0.509		
Adjusted R^2^	0.499		
Residual Std. Error (df = 190)	34.729		
F-Statistic (df = 4; 190)	49.254 ***		

BMI, age (months) and step counts were entered as continuous variables, sex was entered as a dichotomous variable (1 = boy; 2 = girl); ** *p* < 0.01, *** *p* < 0.001; Abbreviation: BMI, Body Mass Index; df, degrees of freedom; R^2^, the coefficient of determination.

**Table 3:** Results of relationship between the fitness total Z-score, subtests according to endurance, strength, speed, coordination and flexibility, and the cognitive variables.

Cognitive Tasks	Total Z-Score	Speed	Endurance	Flexibility	Coordination	Strength
**Switch**						
Switch costs	ns	ns	ns	ns	ns	ns
RT Visual search letters	ns	ns	ns	ns	−0.179 (0.011)	ns
RT Visual search numbers	−0.173 (0.014)	−0.154 (0.029)	ns	ns	−0.241 (0.001)	ns
RT Switch	ns	ns	ns	ns	−0.205 (0.004)	ns
**Corsi block-tapping**						
Longest Path	0.191 (0.005)	0.154(0.025)	0.152 (0.027)	ns	0.171 (0.013)	0.153 (0.026)
Correct immediate block span	0.227 (0.001)	0.234 (0.001)	0.146 (0.033)	ns	0.259 (<0.001)	0.187 (0.006)
Score	0.230 (0.001)	0.225 (0.001)	0.157 (0.022)	ns	0.253 (<0.001)	0.189 (0.006)
**2-back**						
Ratio of false alarms	−0.188 (0.006)	ns	−0.174 (0.012)	−0.175 (0.011)	−0.181 (0.009)	−0.161 (0.019)
RT	−0.182 (0.008)	−0.172 (0.013)	ns	ns	−0.232 (0.001)	ns
Count of correct events	0.186 (0.007)	ns	0.169 (0.014)	0.204 (0.003)	0.185 (0.007)	0.157 (0.023)
**Flanker**						
RT True-komp	−0.232 (0.001)	−0.242 (<0.001)	ns	ns	−0.272 (<0.001)	−0.203 (0.003)
RT True-Ikomp	−0.189 (0.006)	−0.208 (0.002)	ns	ns	−0.239 (<0.001)	−0.146 (0.034)
Ratio False Komp	ns	ns	ns	ns	ns	ns
Ratio False IK	ns	ns	ns	ns	ns	ns
Count of false alarms	ns	ns	ns	ns	ns	ns

Data are presented as a Spearman’s rank correlation coefficient (*p*-value); Total Z-score comprises results of eight subtests according to speed (20 m sprint), endurance (6 min run), flexibility (forward bending), coordination (lateral jumping and balancing backwards) and strength (sit-ups, standing long jump and push-ups). Abbreviations: RT, reaction time; ns, not significant.

**Table 4:** Multivariable analysis of the associations between the fitness skills and the cognitive outcomes considering other predictors estimated by backwards regression.

Task	*p*-Value	Adjusted R^2^	Descending Order of the Predictors Based on the Standardized Coefficients (*β* coefficient)
**Switch**			
Switch costs	ns		
RT Visual search letters	0.007	0.055	total Z-score (−952.8 *), strength (632.5), steps 8–13 (−3.985 *), sex (−4303.6)
RT Visual search numbers	<0.001	0.081	coordination (−341.6 **), age (−261.6 *), PE class (−4008.6), sex (4073.3)
RT Switch	<0.001	0.093	coordination (−810.7 ***), age (−514.1 *), steps 8–13 (−5.7 *), sex (−9172.2 *)
**Corsi block-tapping**			
Longest Path	<0.001	0.107	BMI (0.064 *), total Z-score (0.028), PE class (−0.4), steps 8–13 (0.0002 *)
Correct immediate block span	<0.001	0.085	total Z-score (0.070 **), BMI (0.120 *), sex (−0.838 *)
Score	<0.001	0.094	total Z-score (0.183 **), BMI (0.313 *), sex (−2.319 **)
**2-back**			
Ratio of false alarms	0.005	0.055	sex (−8.5 *), PE class (7.4 *), steps 8–13 (−0.004 *), flexibility (−0.2)
RT	0.009	0.038	steps 8–13 (−0.02 *), total Z-score −1.7)
Count of correct events	0.002	0.067	sex (8.059 **), PE class (−6.901 *), steps 8–13 (0.004 *), flexibility (0.229*)
**Flanker**			
RT Cong	<0.001	0.154	sex (55.9 ***), coordination (−2.3 **), PE class (−37.3 *)
RT ICong	<0.001	0.186	sex (99.5 ***), PE class (−41.2 *), coordination (−1.7), age (1.6), steps 24 h (0.005)
Ratio False Cong	0.006	0.042	age (−0.3 *), sex (−5.1 *)
Ratio False ICong	<0.001	0.116	sex (−8.1 ***), age (−0.4 **), PE class (7.2 *), steps 24 h (−0.001 **), total Z-score (0.3)
Count of false alarms	<0.001	0.168	sex (−8.0 ***), age (−0.2 ***), steps 8–13 (−0.002 *), PE class (3.1 *)

Predictors: DMT total Z-score, speed, endurance, coordination, flexibility, strength, BMI, age (months) and step counts (24 h and 8–13) were entered as continuous variables, PE class and sex were entered as a dichotomous variable (s-PE = 1; r-PE = 2), (1 = boy; 2 = girl); significance level: * *p* ≤ 0.05, ** *p* < 0.01, *** *p* < 0.001; BMI, Body Mass Index; PE, physical education, RT, reaction time.

## Data Availability

Data are contained within the article.
